# Effects of a Novel Pharmacologic Inhibitor of Myeloperoxidase in a Mouse Atherosclerosis Model

**DOI:** 10.1371/journal.pone.0050767

**Published:** 2012-12-10

**Authors:** Cuiqing Liu, Rajagopal Desikan, Zhekang Ying, Liubov Gushchina, Thomas Kampfrath, Jeffrey Deiuliis, Aixia Wang, Xiaohua Xu, Jixin Zhong, Xiaoquan Rao, Qinghua Sun, Andrei Maiseyeu, Sampath Parthasarathy, Sanjay Rajagopalan

**Affiliations:** 1 Department of Physiology, Hangzhou Normal University, Hangzhou, China; 2 Davis Heart and Lung Research Institute, The Ohio State University, Columbus, Ohio, United States of America; 3 InVasc Therapeutics, Tucker, Georgia, United States of America; Maastricht University, The Netherlands

## Abstract

Inflammation and oxidative stress play fundamental roles in the pathogenesis of atherosclerosis. Myeloperoxidase has been extensively implicated as a key mediator of inflammatory and redox-dependent processes in atherosclerosis. However, the effect of synthetic myeloperoxidase inhibitors on atherosclerosis has been insufficiently studied. In this study, ApoE^−/−^ mice were randomized to low- and high-dose INV-315 groups for 16 weeks on high-fat diet. INV-315 resulted in reduced plaque burden and improved endothelial function in response to acetylcholine. These effects occurred without adverse events or changes in body weight or blood pressure. INV-315 treatment resulted in a decrease in iNOS gene expression, superoxide production and nitrotyrosine content in the aorta. Circulating IL-6 and inflammatory CD11b^+^/Ly6G^low^/7/4^hi^ monocytes were significantly decreased in response to INV-315 treatment. Acute pretreatment with INV-315 blocked TNFα-mediated leukocyte adhesion in cremasteric venules and inhibited myeloperoxidase activity. Cholesterol efflux was significantly increased by high-dose INV-315 via ex-vivo reverse cholesterol transport assays. Our results suggest that myeloperoxidase inhibition may exert anti-atherosclerotic effects via inhibition of oxidative stress and enhancement of cholesterol efflux. These findings demonstrate a role for pharmacologic modulation of myeloperoxidase in atherosclerosis.

## Introduction

Myeloperoxidase (MPO) is a hemoprotein produced by polymorphonuclear neutrophils and macrophages and is thought to play a role in atherosclerosis through its role in inflammation and oxidative modification of low-density lipoprotein (LDL) and high-density lipoprotein (HDL) [1]–[3]. MPO is released during inflammatory activation of the immune cells [2] and contributes to not only events integral to the inception of plaque but also processes that may confer plaque vulnerability [4], [5]. MPO is present in human atherosclerotic areas rich in macrophages and consistent with its role, mass spectrometric approaches reveal lipid and protein oxidation products characteristic of its peroxidase function [2], [6]. MPO-dependent nitration of amino acid residues such as tyrosine has been linked to altered protein structure and function of lipoproteins. For example, MPO-modified HDL impairs its ability to partake in reverse cholesterol transport (RCT) [7], [8]. Collectively, these observations provide strong evidence that MPO is present and enzymatically active in atherosclerotic tissue.

The pathophysiologic role of MPO in cardiovascular disease has attracted considerable interest in the development of MPO inhibitors for therapeutic use. To our knowledge, safe and efficacious MPO inhibitors are still lacking currently, although Azide, 4-aminobenzoic acid hydrazide (4-ABAH) has been used as a MPO inhibitor for a long time [9]. We recently synthesized a novel small molecule inhibitor of MPO, INV-315, and investigated its pharmacokinetics, safety and efficacy in a model of atherosclerosis. Here we demonstrate that a small molecule approach towards MPO inhibition is feasible and effective in reducing atherosclerosis and improving vascular function via attenuation of inflammation, oxidative stress and enhancement of cholesterol efflux.

## Methods

### Animal model

Twenty-seven male ApoE^−/−^mice (4 weeks of age, n = 9 for each group) were purchased from Jackson Laboratories (Bar Harbor, ME) and were allowed to equilibrate for 2 weeks before being fed high-fat diet (HFD) containing 42% of calories from fat (TD.88137, Harlan, Madison, WI) or HFD admixed with INV-315 2 mg/kg/day (low-dose group) or 10 mg/kg/day (high-dose group) for 16 weeks. All mice were maintained at 21°C on a 12-h light/12-h dark cycle with free access to water and food. All procedures of this study were approved by the Committees on Use and Care of Animals and the office of Responsible Research Practics, Human Institutional Review Board of The Ohio State University (Protocol Approval #2009A0195, #2008H0177). Human informed consent was obtained in writing and a copy was inserted in the medical record of the patients.

### Measurement of blood pressure, metabolic parameters, lipoproteins and circulating cytokines levels

The time line of events of the treatment protocol was sketched as shown in Figure S1. One week before the end of the experiment, blood pressure and pulse were measured in conscious mice using a computerized non-invasive tail-cuff manometry system (Visitech IITC model 129 system, Visitech Systems, Apex, NC). Mean blood pressure (MBP) and pulse were measured each day at the same time, by the same experienced operator for one week. All mice were firstly acclimated to the measurements for several days (these data were discarded) and then the parameters were determined as the average of measurement over 4 days. In addition, during each day, 10 acclimatization cycles were followed by 20 measurement cycles, which were collected to obtain the average values for blood pressure and pulse for each individual mouse for a particular day. At the end of the experiment, mice were fasted overnight and Intra-peritoneal glucose tolerance test (IPGTT) was performed using previously described methods [10]. Just before sacrifice blood will be procured under full isoflurane anesthesia by retro-orbital bleeding, followed by euthanasia. Plasma was collected after the whole blood centrifuging at 500 *g*, 4°C for 5 minutes. 100 µl plasma was used for profile of plasma lipoproteins [HDL, cholesterol and triglyceride (TG)] [11] by Cardiovascular Specialty Laboratories, Inc (Atlanta, GA). Circulating cytokine levels were determined by Cytometric Bead Array (BD Biosciences, San Diego, CA). 50 µl Plasma was incubated with beads specific for interferon γ (IFN-γ), monocyte chemoattractant protein 1 (MCP-1), interleukin 6 (IL-6), and IL-10 according to the manufacturer's instructions. The total amount of cytokines was then determined using a BD LSR II instrument and analyzed by the BD CBA software (BD Biosciences).

### Functional vascular assessment and quantification of atherosclerosis

Functional vascular assessment was performed as previously described [12], [13].

The aortic root and adjacent heart were embedded in Optimum Cutting Temperature (OCT) and 10-µm thick sections were obtained from the annulus extending through the aortic sinus region. Sections were stained with haematoxylin and eosin (H&E) or Masson's trichrome. Atherosclerotic quantification was performed as described previously [14].

### Localization/quantification of nitrotyrosine by immunohistochemistry and superoxide anion by dihydroethidium

These methods are described in Methods and Results S1.

### Monocyte subset assessment by flow cytometry

Spleens were isolated, homogenized and suspended in phosphate buffered saline (PBS). Bone marrow derived cells were collected by flushing the femur and tibia with PBS. These cells were centrifuged at 500 *g* for 5 minutes. Whole blood was centrifuged at 500 *g*, 4°C for 5 minutes and plasma was collected. The remaining blood cells and the resulting pellet of splenic and bone marrow derived cells were re-suspended in 1× red blood cell lysis buffer (Biolegend), at room temperature for 3 min followed by the addition of PBS and centrifugation. Then, cells were stained with anti-CD11b, anti-7/4, anti-Ly6G followed by incubation at room temperature for 45 min. Cells were subsequently washed with PBS and re-suspended in 1% neutral buffered formalin and run by flow cytometry (BD FACS LSR II™ flow cytometer, Becton Dickinson, San Jose, CA). Data was analyzed using BD FACS Diva software (Becton Dickinson, San Jose,CA). The antibodies were purchased from Biolegend, Miltenyi Biotec, or BD Bioscience.

### Quantitative RT-PCR

RNA was extracted from tissues including thoracic aorta, small intestine and liver with Trizol (Invitrogen, Carlsbad, CA, USA) and CD11b^+^ cells from bone marrow with an Absolutely RNA MiniPrep kit (Stratagene, La Jolla, CA, USA) following the manufacturer's instructions. cDNA was reversely transcribed using High Capacity cDNA Transcription kit (Applied Biosystems, Carlsbad, California, USA). Quantitative polymerase chain reaction (qPCR) was performed in duplicate using Lightcycler 480 (Roche). “The expression level for each gene was calculated using the ΔCt method relative to β-actin. The sequences of all primers used are listed in Table S1.

### Measurement of cholesterol efflux

At the end of the experiment, ∼0.1 ml blood was collected via tail bleed after mice had been fasted overnight. Serum was harvested and stored frozen at −20°C until further use. HDL-enriched serum fractions were isolated after treatment of serum samples with HDL precipitation buffer (Abcam, Cambridge, MA) according to manufacturer instruction. J774 cells were cultured as described elsewhere [15] and seeded in 24-well plates at a density of 5×10^5^ cells/well in serum-free Dulbecco's Modified Eagle Medium (DMEM). Next, cells were labeled overnight with acLDL probe [16] containing [1α2α(n)-^3^H]-cholesterol (American Radiolabeled Chemicals, Inc., St. Louis, MO) at 1 µCi/ml. Cells were washed with phosphate-buffered saline (3×1 ml), and further incubated in DMEM containing the test HDL at a final concentration of 2.5% for 4 h at 37°C. Medium and cells were collected, and aliquots taken for counting of radioactivity. Cholesterol efflux was determined as the proportion of radioactivity in medium divided by radioactivity in medium plus cells. Background efflux, where efflux to medium without tested HDL, was subtracted [16].

### Leukocyte trafficking by intra-vital microscopy

To test the effect of INV-315 on acute inflammation, C57BL/6 mice were injected with INV-315 (100 mg/kg, determined by preliminary experiments) or equal volume of vehicle as placebo, then administered TNFα at a dose of 1 µg/kg [17]. After 4 hours, mice were anesthetized by a mixture of ketamine (100 mg/kg) andxylazine (20 mg/kg). All drugs were administered intra-peritoneally. Cremasteric muscle was exteriorized, mounted on aplexiglas platform, and superfused with pre-warmed Ringer's lactate (37°C). The number of the rolling cells per 30 seconds per image field (1.57×10^5^ µm^2^) was counted, and cells that remained stationary for the whole 30 seconds were considered “adherent” cells [17]. The data presented were averaged from 5–10 vessels per mouse. Metamorph software (version 7.1.2.0, Metamorph, Downingtown, PA) was used for analysis of events.

### Myeloperoxidase activity analysis

MPO activity was analyzed in peritoneal cavity macrophages and human peripheral blood using 2 different approaches. MPO activity in periteonal macrophages was assessed with the H_2_O_2_-dependent tetramethylbenzedine (TMB) oxidation assay at 650 nm [18]. 1×10^5^ peritoneal macrophages were used per assay. For human peripheral blood MPO activity, a luminol-based substrate [19] was used using a 96-well plate. Heparinized whole blood (1 µl) freshly isolated from healthy volunteers was diluted in 200 µl of modified Hank's Buffered Salt Solution (HBSS) containing luminal (100 µM) and fetal bovine serum (FBS) (1%, vol/vol) in the absence or presence of increasing concentrations of INV-315 (0.1–100 µM) or 4-ABAH (5 µM). Samples were read or imaged before (*t* = 0) and at the indicated time points after stimulation with phorbol 12-myristate 13-acetate (PMA, 5 µM) or same volume of vehicle (dimethyl sulfoxide). Luminescence signal was recorded on a Berthold luminometer (Berthold technologies, Oak Ridge, TN, USA) and also detected on a IVIS Xenogen bioluminescence imager (Caliper LifeSciences, Hopkinton, MA).

### Data analysis

Data are means ± standard error of the mean for the number of animals indicated. Graphpad Prism software (Version 5) was used for one-way ANOVA and Bonferroni's *post*-hoc test where appropriate. Value of EC_50_ stands for the concentration needed to cause 50% of the maximal effect as determined by non-linear regression curve fitting. Concentration-relaxation curves were analyzed by two-way ANOVA followed by Bonferroni's *post*-tests. *P* value of <0.05 was considered statistically significant.

## Results

### *In-vitro* MPO inhibition and pharmacokinetics

INV-315 met Lipinski's criteria for drug likeness and was selected from several candidate molecules based on in-vitro assays of MPO inhibition (manuscript submitted and under review). Tables S2, S3, S4, S5, and S6 provide aqueous solubility, metabolic stability and toxicity data. Previously performed experiments demonstrated efficacy in inhibition of MPO activity (IC_50_ = 0.9 µM). *In vitro* assays to MPO inhibition demonstrated efficacy in inhibition of MPO activity (IC_50_ = 0.9 µM). Figure S2 depicts plasma PK, with the half life of the molecule as 42±4 min with oral administration (5 mg kg^−1^) and 119±84 min with IV administration (1 mg kg^−1^) (Table S6).

### Effects of MPO inhibition on metabolic parameters

There were no differences in body weight between the groups at baseline. 16-week of HFD feeding resulted in significant increase in body weight without significant effects between control and INV-315-treated groups at the end of the treatment period (Table S7). Intra-peritoneal glucose tolerance tests showed that treatment with INV-315 had no effects on plasma glucose over time, reflected by the area under the curve. A trend towards a drop in MBP and a probable compensatory rise in pulse at the end of treatment period were observed (Table S7). There were no differences in plasma HDL and total cholesterol, although there was a trend towards reduction in TG, in the high-dose group (Table S7).

### Chronic MPO inhibition results in reduced plaque burden

**Figure 1:**
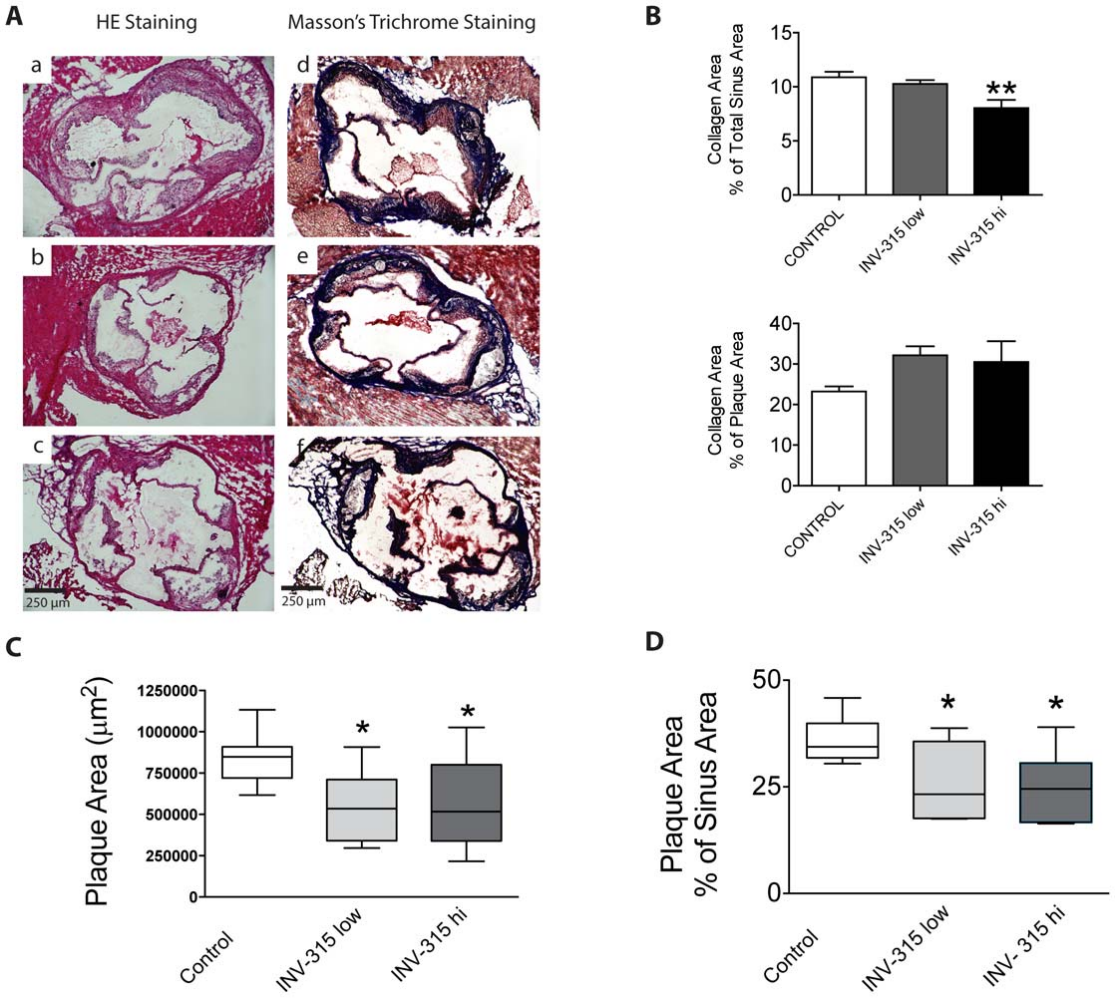
Effects of MPO inhibition on atherosclerosis in ApoE^−/−^ mice fed on HFD. A. Images of aortic sinus stained with H&E staining and Masson's trichrome staining from HFD fed ApoE^−/−^ mice treated with placebo (a and d) as control or low dose (b and e) or high dose (c and f) of INV-315. B. Collagen content in plaque in 3 groups by Masson-trichrome staining, expressed as percent of collagen area relative to total sinus area or plaque area. Data are mean± S.E.M. C and D. Box plot of plaque burden quantified by absolute plaque area (C) and percent of plaque area relative to sinus area (D). The box represents the upper and lower quartiles. The whiskers show the 25 and 75 percentiles, and the line in the box represents the median. *P*<0.05, ** *P*<0.01 compared with control group. Data from 7–9 different mice.

Figure 1A depicts a representative micrograph of plaque burden at the level of the aortic sinuses. Compared with HFD-fed control group, INV-315 decreased plaque burden (26±4%, 25±3% and 36±2% in low, high and control groups respectively, *P*<0.05 for both dose groups vs. control, Figure 1C–D). This reduction was associated with a parallel decline in plaque collagen when analyzed as percent of collagen area relative to total sinus area, but an increase in collagen content when expressed as the percent of collagen area relative to plaque area (Figure 1A and Figure 1B).

### Chronic MPO inhibition improves endothelial function

**Figure 2:**
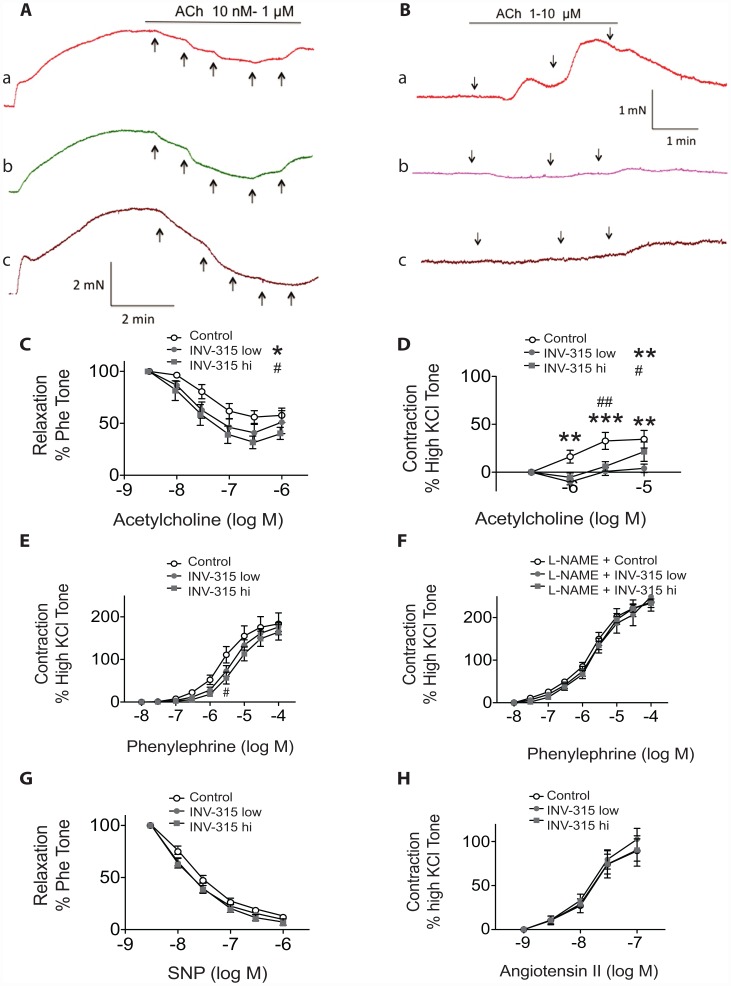
Effect of MPO inhibition on vascular dysfunction in ApoE^−/−^ mice fed on HFD. A and B. Representative traces showing acetylcholine (ACh)-induced relaxations in phenylephrine (Phe)-contracted abdominal arterial rings (A) and ACh-induced contractions of abdominal arterial rings in presence of L-NAME (B) from HFD fed ApoE^−/−^ mice treated with placebo as control (a) or low dose (b) or high dose (c) of MPOi. C–H, Concentration-response curves in abdominal arterial rings for ACh without (C) and with (D) L-NAME treatment, phenylephrine with (F) and without (E) L-NAME treatment, SNP (G) and Angiotensin II (H). *P<0.05, **P<0.01, *** *P*<0.001 indicates significant difference when INV-315 low dose group compared with control group or compared with control group at respective concentration; #*P*<0.05, ##*P*<0.01 indicates significant difference when INV-315 high dose group compared with control group or compared with control group at respective concentration. Data are mean± S.E.M. from 5 different mice.

Traces in Figure 2A show that acetylcholine caused a concentration-dependent relaxation of abdominal aorta rings pre-constricted with phenylephrine. INV-315 treatment resulted in an improvement in acetylcholine-induced relaxation of aortic segments (Figure 2C). In the presence of NG-nitro-L-arginine methyl ester (L-NAME) at 100 µM, acetylcholine elicited pronounced contraction of aortic rings, with a maximal response of ∼1.4±0.1 mN (Figure 2B), corresponding to 34.4±9.2% of the contractile response induced by 120 mmol/L KCl (Figure 2D). The acetylcholine induced contraction was attenuated in rings from mice fed on HFD with INV-315 at low dose and high dose, *P*<0.05 compared with control (Figure 2D). Table S8 depicts the EC_50_ values and % maximal response (Emax) to the various interventions. Figure 2E, depicts a shift in dose response to phenylephrine at concentration of 3 µM, which was abolished with L-NAME pretreatment (Figure 2F). MPO inhibition shows no alteration in sodium nitroprusside (SNP)-induced relaxation (Figure 2G) or Angiotensin II-induced vascular contraction (Figure 2H).

### Chronic MPO inhibition decreases o_2_^•−^ production and nitrotyrosine formation

**Figure 3:**
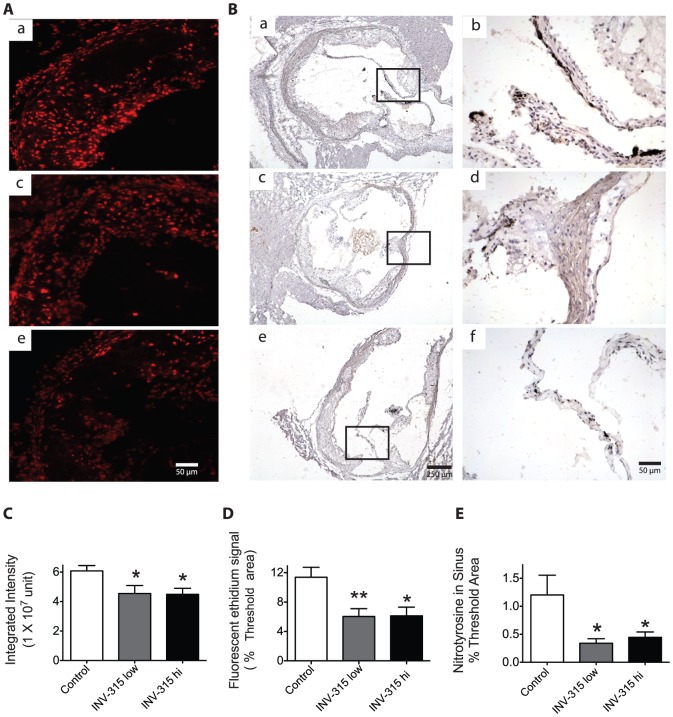
Effects of MPO inhibition on superoxide and nitrotyrosine within the mice sinus of HFD-fed mice. A. Fluorescent photomicrographs at identical settings of sections of sinus labeled with dihydroethidium [1] at 200× magnification. a, control mice treated with placebo; b, mice treated with INV-315 at low dose; c, mice treated with INV-315 at high dose. B, Representative images of immunohistochemistry for nitrotyrosine in the sinus. a and b, control mice treated with placebo; c and d, mice treated with INV-315 at low dose; e and f, mice treated with INV-315 at high dose. b, d and f in the right panel are higher magnifications of a, c and e in the left panel respectively. C and D. Quantification of the fluorescent ethidium signal in the sinus by integrated intensity (C) and % threshold area (D). E. Statistical analysis of immunohistochemistry for nitrotyrosine in the sinus. *P<0.05, **P<0.01 compared with control group. Data are mean± S.E.M. of 7–9 experiments from different mice.

Figure 3 shows result of DHE staining and immunohistochemistry for superoxide and nitrotyrosine level measurement. Quantification of the fluorescent signal showed a ∼1.9-fold decrease in O_2_^•−^ Production shown by fluorescence in the aorta in both INV-315 treated groups compared to control fed HFD only (Figure 3A, 3C, 3D, *P*<0.05 for both groups vs. control). INV-315 treatment resulted in 2–3.5-fold decrease in nitrotyrosine content in the aortic sinus (Figure 3B, 3E).

### Chronic MPO inhibition alters inflammation but not reverse cholesterol transport (RCT) gene expression

**Figure 4:**
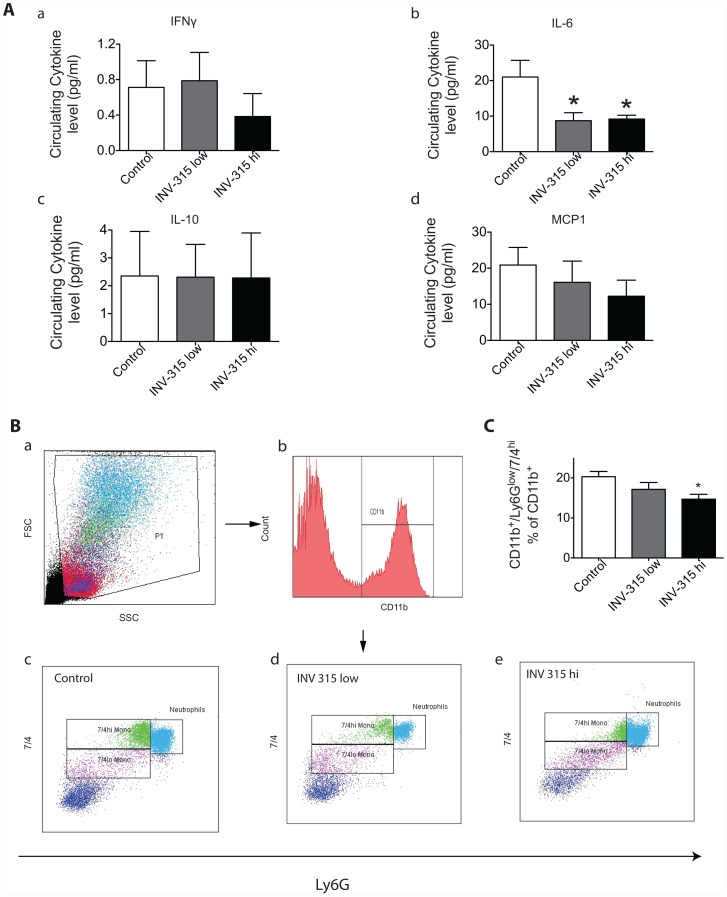
Effects of MPO inhibition on inflammatory cytokines and monocytes in blood from HFD-fed ApoE^−/−^ mice. A. The concentrations of (IFN-g, IL-6, IL-10 and MCP-1) in response to INV-315 in ApoE^−/−^ mice fed on HFD. B. Representative flow-cytometric dot plots showing circulating CD11b^+^Ly6G^low^7/4^hi^cells from mice blood. C. Flow cytometric analysis of inflammatory cells stained for CD11b^+^Ly6G^low^7/4^hi^. *P<0.05 compared with control group. Data are mean± S.E.M. of 7–9 experiments from different mice.

To assess the effect on inflammatory gene expression, we compared the circulating levels of cytokines, and expression of genes encoding pro-inflammatory proteins in thoracic aorta tissue from mice treated with and without INV-315. As shown in Figure 4A, there was no significant difference in IFN-γ, IL-10 or MCP-1 between groups. However, circulating IL-6 level was decreased by INV-315 treatment at both doses. Compared with HFD fed group, we observed a 2–3.5-fold decrease in inducible nitric oxide synthase (*inos*) expression in aorta from mice fed on HFD with INV-315 (Figure S3A). In contrast, there were no changes in *il-6*, tumor necrosis factor-alpha (*tnf-α*) expression or *ccl2*, *ccr2*, *ccl5* or *ccr5* (Figure S3B, S3C, S3E–S3H). *mpo* expression was itself not altered by (Figure S3D) MPO inhibition. INV-315 treatment induced no changes in the expression of *abca1*, *abcg1* or *srb1* in the aorta (Figure S4A–S4C).

### Monocyte subsets in response to MPO inhibition

In the present study, we defined monocytes as side scatter-low, forward scatter-high cells expressing the myeloid antigen 7/4 (high populations) and high levels of CD11b but showing no expression for the neutrophil marker Ly6G. The CD11b^+^Ly6G^low^7/4^hi^ cells correspond to Ly6C^hi^ monocytes, representing the inflammatory subtype [20]. Our results showed that INV-315 treated group at high dose significantly reduced the level of circulating CD11b^+^Ly6G^low^7/4^hi^cells (20.3±1.3% in control group, 17.1±1.7% in low dose group and 14.7±1.2% in high dose group, *P*<0.05 for high dose group vs. control group, Figure 4B, 4C). In contrast to its reduction in blood, we did not find any reduction of the inflammatory monocytes in bone marrow and spleen (data not shown).

### MPO inhibition enhances cholesterol efflux

In order to assess the effects on inflammation, a PCR array was utilized to profile the expression of *il-6*, *tnfa* and *ccl2* genes in liver, bone marrow-derived monocytes and small intestine. We found no significant difference of the 3 pro-inflammatory genes expression in these tissues and monocytes (Figure S5). Neither was RCT-related gene altered by INV-315 treatment, Figure S3D–S3O. However, INV-315 treatment increased cholesterol efflux from macrophages at high dose, compared to HFD fed control (*P*<0.05, Figure S5A), indicating improved RCT function of HDL.

### Acute effect of MPO inhibition on leukocyte trafficking in microcirculation

**Figure 5:**
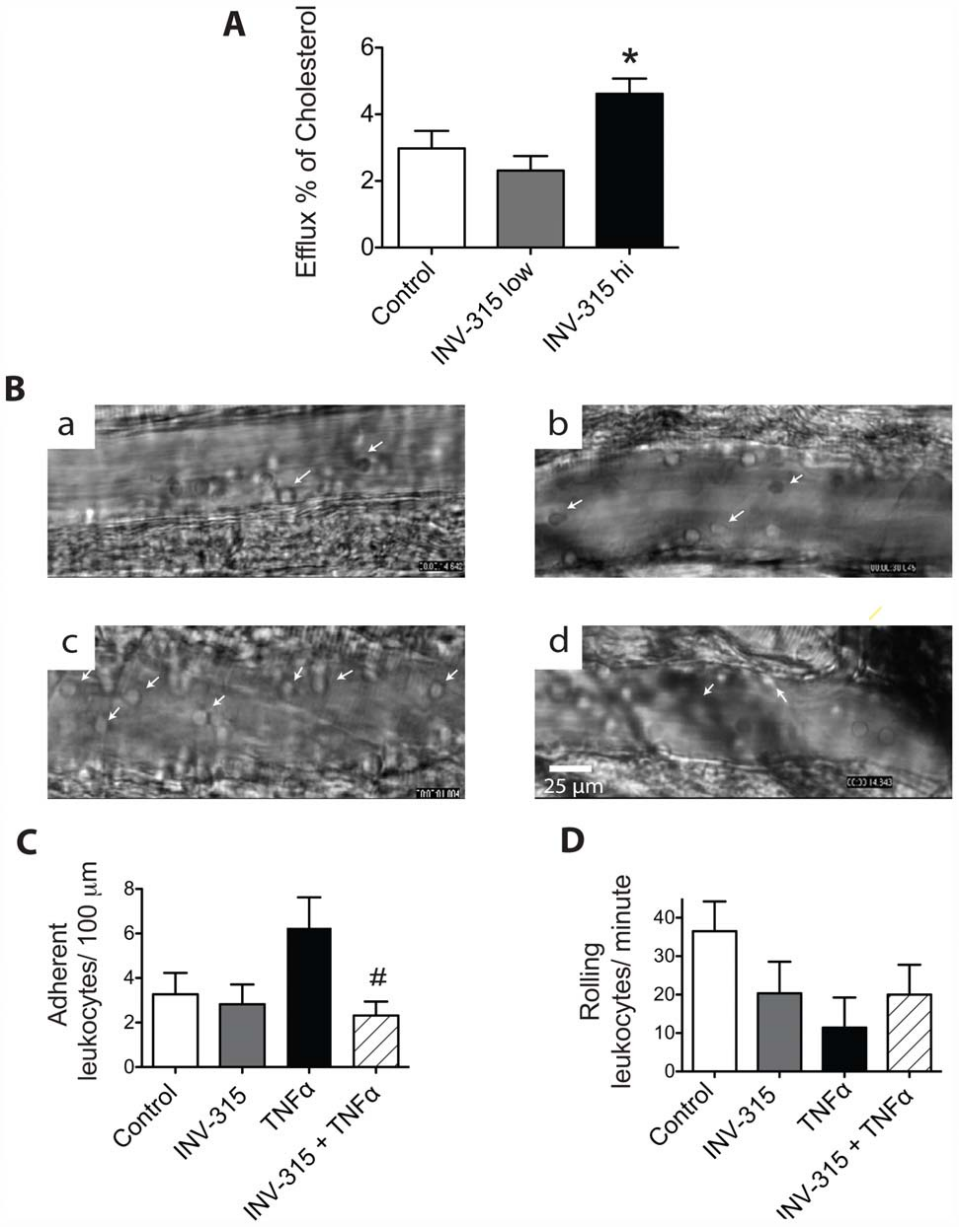
Effects of MPO inhibition on HDL transporting cholesterol and leukocyte adhesion/rolling flux. A. Effects of MPO inhibition on HDL transporting cholesterol from J774 macrophage cells into medium. *P<0.05, compared with control group. Data are mean± S.E.M. of 5–8 experiments from different mice. B. Representative images of adherent leukocytes in the cremasteric microcirculation (cells with arrow heads indicate adherent monocytes, cells without arrow heads indicate rolling leukocytes) via intra-vital microscopy in C57BL/6 mice with different treatment. a, blank control group; b, INV-315treated control; c, TNFα-treated group; d, TNFα-treated groups with INV-315 pretreatment. C and D, Leukocyte adhesion and leukocyte rolling flux in cremasteric microcirculation via intra-vital microscopy in C57BL/6 mice with different treatment. #*P*<0.05 compared with TNFα treated group. Data are mean ± SD of 5 different mice, 5–10 experiments from each animal.

In order to further evaluate the significance of the role of MPO inhibition in inflammation, we conducted acute experiments on C57BL/6 mice that were treated with INV-315 (100 mg/kg) or vehicle, followed by TNFα. TNFα intra-peritoneal injection resulted in an increase in adherent monocytes and decrease in rolling leukocytes in the microcirculation when compared with untreated control mice (Figure 5B-a, 5B-c, Figure 5C). The enhanced adherence of leukocytes in the TNFα-treated group was decreased by pretreatment with INV-315 (Figure 5B-d, Figure 5C). The drug itself in the absence of TNFα had no effect on the number adherent leukocytes (Figure 5B-b). Conversely, the number of rolling monocytes in response to TNFα injection was decreased, likely related to increased adherence, an effect that was reversed by MPO inhibition pretreatment (Figure 5D).

### Effects of INV-315 on MPO activity

**Figure 6:**
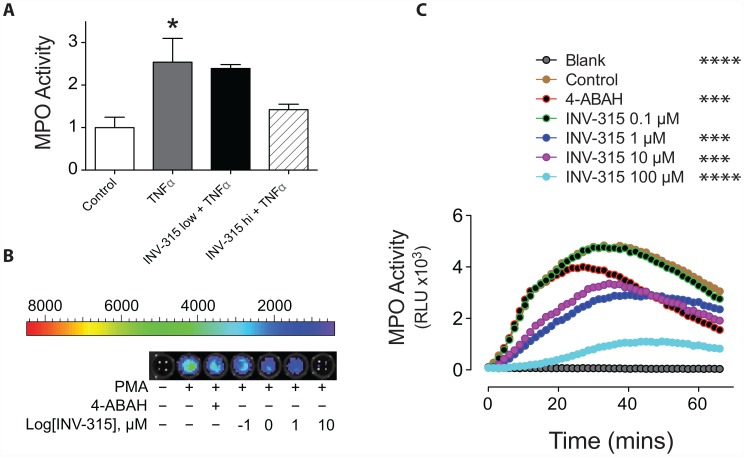
Effect of INV-315 on MPO activity in mice peritoneal macrophages and human blood. A. MPO activity in macrophages derived from the peritoneal cavity of C57BL/6 mice. Data were derived from 5–7 mice for each group. B. Luminol-bioluminescence imaging of PMA-stimulated blood. C. Luminol bioluminescence was plotted as a function of time and concentration of INV-315. Data were derived from triplicate results. *P<0.05, **P<0.01, ***P<0.001, ****P<0.0001, compared with control group.

Since MPO was also identified in mice peritoneal macrophages [21], we additionally confirmed effects on MPO activity in separate ex-vivo experiments with mice macrophages and human peripheral blood. TNFα induced increase in MPO activity in mice peritoneal cavity macrophages was attenuated by pre-administration of INV-315 dose-dependently (10 mg/kg and 100 mg/kg) when compared with vehicle plus TNFα treatment group (Figure 6A). In experiment with human blood, the whole blood incubated with luminol was treated with the potent protein kinase C activator PMA or vehicle control (Figure 6B, 6C). PMA induced a time-dependent increase in bioluminescence, peaking approximately 25–35 minutes after stimulation (Figure 6C), which was inhibited by 4-ABAH, a commercial MPO inhibitor pretreatment (Figure 6B, 6C). INV-315, dose-dependently inhibited the increase in luminescence signal, with effects that were superior to 4-ABAH (Figure 6B, 6C). No significant change in bioluminescence was observed in unstimulated cells (Figure 6B, 6C).

## Discussion

This work has multiple important findings that support a small molecule strategy to inhibit MPO, a protein that has been extensively implicated in atherosclerosis: (1) Dietary administration with a small molecule inhibitor of MPO, INV-315 decreased atherosclerotic plaque burden and a reduction in inflammation. (2) This was paralleled by improvements in endothelial function, decreased oxidative stress and nitrotyrosine formation. (3) An effect on reduced activation of inflammatory cells was evidenced by inhibition of leukocyte adhesion acutely and by reduced CD11b^+^/Ly6G^low^/7/4^hi^ monocytes with chronic treatment. (4) MPO inhibition enhanced *ex-vivo* reverse cholesterol transport. These findings provide strong mechanistic rationale for the use of small molecule to inhibit MPO in experimental atherosclerosis.

MPO, a 140-kDa heme-containing homo-dimer [22], is stored in primary azurophilic granules of leukocytes and secreted into both the extracellular milieu and the phagolysosomal compartment following phagocyte activation by a variety of agonists [2]. Our results demonstrate favorable effects on lesion formation that occurred in the absence of overt safety, metabolic or hemodynamic effects suggesting a rather specific effect in reducing plaque burden. MPO oxidizes the NO-metabolite NO_2_^−^, which is produced in areas of inflammation, forming a reactive nitrogen species, presumably nitrogen dioxide (NO_2_) [23], [24]. In addition, NO_2_^−^ can be oxidized by MPO-generated HOCl, forming NO_2_Cl [24]. These reactions then mediate nitration of free and protein-associated tyrosine residues to 3-NO_2_Tyr [23]–[25], which is critically linked to altered protein structure and function during inflammatory conditions [26]. Reduced nitrotyrosine formation in aorta in response to INV-315 treatment in our experiments, is consistent with an effect of MPO inhibitor on this process. Chronic administration of INV-315 was also associated with a reduction in CD11b^+^ Ly6G^low^ 7/4^hi^ monocytes. This subset is believed to mediate pro-inflammatory effects in atherosclerosis and decrease in this subset has been associated with favorable end-points including regression of atherosclerotic lesions and macrophage accumulation [20]. Reduction in adherence of inflammatory leukocytes in response to TNFα as shown by intra-vital microscopy is additional evidence for a direct effect of MPO inhibition in preventing the activation state of these cells. Taken together with a reduction in IL-6, these results demonstrate a beneficial role of chronic MPO inhibition on inflammation in atherosclerosis.

The improvements in endothelium function observed by us may represent a consequence of favorable effect on plaque progression. Moreover, reduced superoxide generation and decreased iNOS expression in response to INV-315 treatment may also help improve endothelium function by decreasing ONOO^−^ formation. In addition, one may speculate direct effects of MPO inhibition on redox chemistry. For instance, MPO may mediate consumption of NO via radical species [27] or through oxidization of NO_2_^−^ to the reactive species NO_2_^•^, which in turn may affect nitration protein-associated tyrosine residues to 3-NO_2_Tyr [23], [24]. This product is critically linked to altered protein structure and function during inflammatory conditions [26]. Thus, the interruption of NO consumption or NO_2_^•^ generation may have resulted in a favorable effect on NO mediated responses in the vasculature observed in our results. In addition, the marginal trend towards reduction in MBP may likely represent a cause or consequence of the improvements in endothelial function.

HDL has been proposed to lose its cardio-protective effects in subjects with atherosclerosis, which involves oxidative damage by MPO. Our data showed no significant alteration of RCT genes [ATP binding cassette (ABC) transporters] in liver, small intestine and bone marrow-derived monocytes with chronic administration of INV-315. Ex-vivo reverse cholesterol transport assays demonstrated an improvement in cholesterol efflux in response to HDL from INV-315 treated mice. Since MPO-oxidized apolipoprotein A-I (apoA-I) impairs the cellular cholesterol efflux by ABCA1 [3], INV-315 may retard atherosclerosis development via inhibition of HDL oxidation. Bergt's lab identified a single tyrosine residue, Tyr192, as the major site of nitration and chlorination when HOCl oxidizes apoA-I [7], [8] and noted a strong association between the extent of Tyr192 cholorination (but not nitration) and loss of ABCA1 transport activity (dysfunction of HDL) [8]. Whether INV-315 works on this specific residue in apoA-I requires further investigation.

Although there is a strong pathophysiologic basis to support a role for MPO in human atherosclerosis [1], [2], Brennan et al provided evidence of increased lesion formation in LDL receptor-MPO double knockout mice compared to LDL^−/−^mice [28]. A variety of reasons have been ascribed to these results including the lower activity of murine MPO compared to human MPO and the differences in murine anti-oxidant defense systems and a potential homeostatic role for MPO derived oxidants at least at low concentrations. In keeping with this argument, transgenic human MPO expression in mice correlated with increases in lesion size and lipid profile [29]. These differences notwithstanding, our results are nonetheless important and differ from prior studies involving MPO^−/−^ models. Pharmacologic inhibition of MPO differs from complete absence of MPO in a knock-out model as there are counter-regulatory responses that may be operational in the latter situation. The dose used in our experiments and the half-life of the MPO inhibitor based on PK, would have afforded partial inhibition of MPO for a limited duration of time (t1/2 = 119±84 min).

## Limitations and Conclusions

Our study has multiple important limitations that must be acknowledged. Firstly, MPO has been extensively implicated as a key mediator of lipoprotein oxidation. No evidence of modification of lipoprotein oxidation in response to INV-315 was demonstrated in the present study. We have thus no evidence to support an effect of our compound on these processes as being directly responsible for the salutary effects. INV-315 was admixed and administered through chow in this study, however, the dose levels could vary considerably as compared to oral dosing by gavage. Although this may help to explain the lack of dose dependency, food intake measurement during the treatment period would provide direct evidence. Our assays on RCT have been performed *ex-vivo* and whether these results are an explanation for the observed effects will need careful confirmation in additional studies. Measurement of HDL function *in-vivo* and assessment of alteration in function of MPO targets such as HDL and eNOS (expression, uncoupling or acitivity) may provide further evidence of specificity. Notwithstanding these limitations, our results support small molecule approaches to target MPO in atherosclerosis.

## Supporting Information

Methods and Results S1 (DOCX)

Table S1 **Primers used for real-time PCR.**
(DOC)

Table S2 **Solution Properties.**
(DOC)

Table S3 **In vitro Absorption and Metabolism.**
(DOC)

Table S4 **Drug-Drug Interaction.**
(DOC)

Table S5 **In vitro Toxicity.**
(DOC)

Table S6 **Pharmacokinetic Parameters in Rats venously or orally administered with INV-315.**
(DOC)

Table S7 **Metabolic parameters at the end of the treatment period.**
(DOC)

Table S8 **EC_50_ and Emax (%) values for vascular response.**
(DOC)

Figure S1 **The time line of events of the treatment protocol.**
(TIF)

Figure S2 **Plasma concentrations of INV-315 after intravenously at 1 mg/kg and orally at 5 mg/kg administration.**
(TIF)

Figure S3 **Effects of MPO inhibition on pro-inflammatory gene expression in aorta from ApoE^−/−^ mice fed a HFD.**
(TIF)

Figure S4 **Effects of MPO inhibition on RCT-related gene profiles expression in different tissues from ApoE^−/−^ mice fed a HFD.**
(TIF)

Figure S5 **Effects of MPO inhibition on pro-inflammatory gene expression in different tissues from ApoE^−/−^ mice fed a HFD.**
(TIF)
