# Supplementary material for: Effects of a Novel Pharmacologic Inhibitor of Myeloperoxidase in a Mouse Atherosclerosis Model
Source: PLoS One. 2012 Dec 10;7(12):e50767. doi: 10.1371/journal.pone.0050767 (PMC3519467; doi:10.1371/journal.pone.0050767)
Supplement: Methods and Results S1 — (DOCX) [file pone.0050767.s001.docx]

**Supplemental Methods and Results**

**METHODS**

**Synthesis and Characterization of INV-315**

**Previous studies have demonstrated that ferulic acid, an abundant phenolic phytochemical, found in plant cell wall components may exert important effects on oxidative and nitrosative stress (including MPO based reactions) owing to its phenolic nucleus and extended conjugation[**[**1**](#_ENREF_1)**,**[**2**](#_ENREF_2)**]. Accordingly several ferulic acid derivatives were synthesized initially and validated by in-silico computational docking analysis, by which the mode of binding of these analogues to the active site cavity of MPO was assessed, the strength of protein-ligand interactions was examined and its efficacy was scored accordingly. Based on these initial steps, several analogues were tested using biochemical assays including MPO mediated HOCl generation by HL60 cells. INV-315 was chosen based on favorable PK, PD and additional ADME considerations (unpublished data).**

**Functional Vascular Assessment**

Mice were sacrificed by isoflurane inhalation and cervical dislocation; the whole aorta including the portion contiguous to the heart was dissected out and placed in a dissecting dish filled with ice cold oxygenated Krebs solution (NaCl 119, KCl 4.7, NaHCO_3_ 25, CaCl_2_ 2.5, MgCl_2_ 1, KH_2_PO_4_1.2, and D-glucose 11, all in mM). The abdominal aorta, after careful dissection of adherent connective tissue, was cut into 2 mm-length ring segments. Each ring was suspended between two stainless wires in a 5-ml chamber on a Multi Myograph (Danish, Myo Technology A/S, Denmark) as previously described [[3](#_ENREF_3),[4](#_ENREF_4)]. Krebs solution in the bathing chamber was constantly bubbled with 95% O_2_, 5% CO_2_ and maintained at 37 °C (pH 7.4). Following 60 minutes equilibration, each ring was stretched to 3mN, a determined optimal resting tone for the development of isometric contraction. After artery contractility was assessed with 120 mM KCl, the rings were rinsed in pre-warmed, oxygenated Krebs solution several times until stable resting tone returned and finally equilibrated for 60 min. The resting tone was readjusted to 3mN if necessary. Vascular response to different drugs was then tested.

**Localization and Quantification of Nitrotyrosine by Immunohistochemistry**

Cryosections of mouse sinus were fixed in ice-cold acetone solution for 10 min. The sections were then treated with 0.3% hydrogen peroxide at room temperature for 10 min in order to inhibit endogenous peroxidase activity. After rinsing, the sections were blocked with 1% BSA in phosphate buffered saline (PBS) and then incubated with rabbit anti-mouse nitrotyrosine (Upstate, 1:100) at 4 °C overnight. The sections were incubated with HRP-conjugated goat anti-rabbit IgG (Santa Cruz) at room temperature for 2 hours, and then developed by DAB according to the manufacturer’s instruction (Sigma). Counter-staining on nucleus was performed with haematoxylin. After dehydration, the sections were mounted with Permount®. Negative control was performed in the absence of primary antibody.

**Localization and Quantification of Superoxide Anion by Dihydroethidium**

The oxidative fluorescent probe dihydroethidium (DHE) was used to evaluate in situ O_2_^•-^ production on histological sinus sections of 10-µm thick. DHE is a cell permeable dye that is oxidized by O_2_^•-^ to ethidium bromide, which subsequently intercalates with DNA and is trapped within cell nuclei. DHE 10 µM (Molecular Probes, Invitrogen) was topically applied to each tissue section. Slides were incubated in a light-protected humidified chamber at room temperature for 30 minutes, rinsed with PBS, and analyzed with a fluorescent Nikon Eclipse FN1 microscope (Nikon, Tokyo, Japan). Acquisition settings of the camera were identical for images of ND and HFD specimens. Automatic computer-based analysis was performed using MetaMorph^TM^ software (version 7.1.2.0, Metamorph, Downingtown, PA). Data were expressed as both integrated intensity and % threshold area.

**Pharmacokinetic Assays**

***Absorption, distribution, metabolism, excretion (ADME) and In-vitro Toxicity***: In vitro PK parameters including toxicity were assessed. Briefly, aqueous solubility and partition coefficient were determined by shake-flask technique using HPLC-UV/VIS as a detection method. HPLC-MS/MS was used to test protein binding and apparent permeability coefficient, with the former assessed by equilibrium dialysis technique using human plasma as a source of protein, the latter was determined using a human adeno-carcinoma cell line (Caco-2). The HPLC-MS/MS was also used to measure the metabolic stability of INV-315 in human liver microsomes. In addition, the ability of INV-315 to inhibit permeability glycoprotein (P-gp), an ATP-dependent drug efflux transporter was determined by fluorimetry, using Madin-Darby canine kidney cells expressing the human MDR1 gene (MDR1-MDCKII). Likewise, fluorimetry was used to determine the ability of INV-315 to inhibit different CYP isoforms with appropriate substrates and its effect on cell viability in HepG2 cells using alamar Blue stain.

hERG activity of INV-315 was evaluated by measuring the tail current amplitude with automated whole-cell patch clamp in a subclone of the parental Chinese hamster ovarian cell line (CHO-K1) stably transfected with hERG cDNA.

***In-vivo Pharmacokinetics***: To assess in vivo PK, INV-315 was administered to rats both orally and intravenously, at doses of 5 mg/kg and 1 mg/kg, respectively. Plots of plasma concentration of compound *versus* time were constructed. Parameters included elimination half-life (T1/2), time of maximum observed concentration (Tmax), concentration corresponding to Tmax (Cmax), area under the concentration-time curve from the time of dosing to the time of last (AUC_last_), area under the concentration-time curve from the time of dosing extrapolated to infinity (AUC_inf_), total body clearance (CL), volume of distribution based on the terminal phase (V_z_) and an estimate of the volume of distribution at steady state (V_ss_) obtained from non-compartmental analysis (NCA) of the plasma data using WinNonlin. The bioavailability was calculated based on AUC_inf_ or AUC_last_. Noncompartmental analysis does not require the assumption of a specific compartmental model for either drug or metabolite. NCA allows the application of the trapezoidal rule for measurements of the area under a plasma concentration-time curve [5].

**RESULTS**

**Pharmacokinetics Assay**

As shown in Table S2, in vitro profiling data demonstrated high chromatographic purity and aqueous solubility of INV-315 as high as 99% and 187.4 μM, respectively. Also, Inv-315 exhibited a partition coefficient at index of 2.06 and high protein binding with human plasma proteins (85%) (Table S2). On the other hand, INV-315 showed a low apparent permeability coefficient in Caco-2 cell line (0.5 x 10^-6^ cm/s), with no inhibitory effect on P-gp in MDR1-MDCKII (Table S3). The high metabolic stability (84%) of INV-315 in human liver microsomes indicated low turnover rates in liver (Table S3). Of importance, INV-315 demonstrated little inhibitory effects on different CYP isoforms (Table S4). There was no evidence of in-vitro toxicity even at high doses (100 µM). In hERG CHO-K1 cells, INV-315 at 10 µM obtained 25.1% inhibition on the tail current amplitude (Table S5).

Table S6 shows the in vivo PK parameters of INV- 315 administered orally or intravenously. After I.V injection, The T1/2, CL, Vz, Vss, AUClast and AUCinf were 119 ± 84 min, 26 ± 2 ml/min/kg, 4241 ± 2958 ml/kg, 1335 ± 584 ml/kg, 39454 ± 2978 min*ng/ml and 39660 ± 2996 min*ng/ml, respectively. The concentration of the compound in plasma declined and was almost undetectable 24 h after injection (Table S6). However, after PO administration, INV-315 showed AUClast and AUCinf of 58222 ± 2036 and 58310 ± 2036 min*ng/ml, respectively, with bioavailability of 29 ± 1% (Table S6). The drug peaked in the plasma with a Cmax of 476 ± 4 ng/ml at a Tmax of 30 min, after which it began to decline to 1 ± 0.04 ng/ml 8 h after administration with a T1/2 of 42 ± 4 min (Figure S2 & Table S6).

**REFERENCE**

1 Kato, Y., Nagao, A., Terao, J., and Osawa, T. (2003). Inhibition of myeloperoxidase-catalyzed tyrosylation by phenolic antioxidants in vitro. *Biosci Biotechnol Biochem* **67**, 1136-1139.

2 Kwon, E.Y., Cho, Y.Y., Do, G.M., Kim, H.J., Jeon, S.M., Park, Y.B., Lee, M.K., Min, T.S., and Choi, M.S. (2009). Actions of ferulic acid and vitamin E on prevention of hypercholesterolemia and atherogenic lesion formation in apolipoprotein E-deficient mice. *J Med Food* **12**, 996-1003.

3 Liu, C.Q., Leung, F.P., Wong, S.L., Wong, W.T., Lau, C.W., Lu, L., Yao, X., Yao, T., and Huang, Y. (2009). Thromboxane prostanoid receptor activation impairs endothelial nitric oxide-dependent vasorelaxations: the role of Rho kinase. *Biochemical pharmacology* **78**, 374-381.

4 Sun, Q., Yue, P., Deiuliis, J.A., Lumeng, C.N., Kampfrath, T., Mikolaj, M.B., Cai, Y., Ostrowski, M.C., Lu, B., Parthasarathy, S., Brook, R.D., Moffatt-Bruce, S.D., Chen, L.C., and Rajagopalan, S. (2009). Ambient air pollution exaggerates adipose inflammation and insulin resistance in a mouse model of diet-induced obesity. *Circulation* **119**, 538-546.

5. Gabrielsson, J. and Weiner, D. Pharmacokinetic and Pharmacodynamic Data Analysis: Concepts and Applications. Swedish Pharmaceutical Press. 1997.
