# Supplementary material for: Effects of a Novel Pharmacologic Inhibitor of Myeloperoxidase in a Mouse Atherosclerosis Model
Source: PLoS One. 2012 Dec 10;7(12):e50767. doi: 10.1371/journal.pone.0050767 (PMC3519467; doi:10.1371/journal.pone.0050767)
Supplement: Table S1 — Primers used for real-time PCR. (DOC) [file pone.0050767.s002.doc]

Table S1. Primers used for real-time PCR

| Primer | Forward oligonucleotides | Reverse oligonucleotides |
| --- | --- | --- |
| *Il-6* | 5′-ATCCAGTTGCCTTCTTGGGACTGA-3′ | 5′-TAAGCCTCCGACTTGTGAAGTGGT-3′ |
| *tnf α* | 5′-TTCCGAATTCACTGGAGCCTCGAA-3′ | 5′-TGCACCTCAGGGAAGAATCTGGAA-3′ |
| *inos* | 5′-CTGCTGGTGGTGACAAGCACATTT-3′ | 5′-ATGTCATGAGCAAAGGCGCAGAAC-3′ |
| *Ccl2* | 5′-TCACCTGCTGCTACTCATTCACCA-3′ | 5′-TACAGCTTCTTTGGGACACCTGCT-3′ |
| *Ccl5* | 5′-TCGTGCCCACGTCAAGGAGTATTT -3′ | 5′-TCTTCTCTGGGTTGGCACACACTT-3′ |
| *Abca1* | 5′-ATGTGGAGTTCTTTGCCCTCCTGA-3′ | 5′-CTTTCGTTTGTTGCCGCCACTGTA-3′ |
| *Abcg1* | 5′-TTTGGGCTTACTTTCATGGGCAGC-3′ | 5′-AAGCCAGAAAGCAGGGACTCAGAT-3′ |
| *Abcg5* | 5′-ATGCTAGATGAGCCAACCACAGGA-3′ | 5′-AAGTGTTGGAAGAGCTCAGAGCGA-3′ |
| *Abcg8* | 5′-TTGGACAACCTGTGGATAGTGCCT-3′ | 5′-GGTGAAGTTGCCGATTTGTGTGGT-3′ |
| *Srb1* | 5′-AACACGTACCTCCCAGACATGCTT-3′ | 5′-AGTCCGTTCCATTTGTCCACCAGA-3′ |
| *β-actin* | 5′-TGTGATGGTGGGAATGGGTCAGAA-3′ | 5′-TGTGGTGCCAGATCTTCTCCATGT-3′ |
