# Supplementary material for: Effects of a Novel Pharmacologic Inhibitor of Myeloperoxidase in a Mouse Atherosclerosis Model
Source: PLoS One. 2012 Dec 10;7(12):e50767. doi: 10.1371/journal.pone.0050767 (PMC3519467; doi:10.1371/journal.pone.0050767)
Supplement: Table S2 — Solution Properties. (DOC) [file pone.0050767.s003.doc]

Table S2. Solution Properties

| Assays | Test Concentration (μM) | Results |
| --- | --- | --- |
| Aqueous Solubility * (PBS, pH 7.4; ìM ) | 200 | 187.4 |
| Chromatographic Purity (%) | 200 | 99% |
| Partition Coefficient (log D, n-octanol/PBS, pH 7.4) | 100 | 2.06 |
| Protein Binding* (plasma, human; %) | 10 | 85% |

* Data were obtained from duplicate determinations.
