# Supplementary material for: Effects of a Novel Pharmacologic Inhibitor of Myeloperoxidase in a Mouse Atherosclerosis Model
Source: PLoS One. 2012 Dec 10;7(12):e50767. doi: 10.1371/journal.pone.0050767 (PMC3519467; doi:10.1371/journal.pone.0050767)
Supplement: Table S3 — In vitro Absorption and Metabolism. (DOC) [file pone.0050767.s004.doc]

Table S3. In vitro Absorption and Metabolism

| Assays | Test Concentration (μM) | Results |
| --- | --- | --- |
| A-B Permeability  (Caco-2 cells , pH 6.5/7.4; 10^-6^ cm/s ) | 10 | 0.5 |
|  |  |  |
| P-gp Inhibition  (MDR1-MDCKII, calcein AM substrate; % inhibition) | 1  30  100 | -1.8  -2.8  -0.2 |
| Metabolic Stability  (liver microsomes, human; % parent remaining) | 1 | 84 |

Data were obtained from duplicate determinations.MDR1-MDCKII: Madin-Darby canine kidney cells expressing the human MDR1 gene. Caco-2 cells: Adenocarcinoma cell line.
