# Supplementary material for: Effects of a Novel Pharmacologic Inhibitor of Myeloperoxidase in a Mouse Atherosclerosis Model
Source: PLoS One. 2012 Dec 10;7(12):e50767. doi: 10.1371/journal.pone.0050767 (PMC3519467; doi:10.1371/journal.pone.0050767)
Supplement: Table S4 — Drug-Drug Interaction. (DOC) [file pone.0050767.s005.doc]

Table S4. Drug-Drug Interaction

| Assay | % Inhibition of control values | |
| --- | --- | --- |
|  | INV-315 (10 μM) | INV-315(30 μM) |
| CYP1A2 Inhibition (recombinant, CEC substrate) | - 4 | -6 |
| CYP2B6 Inhibition (recombinant, EFC substrate) | - 8 | -8 |
| CYP2C8 Inhibition (recombinant, DBF substrate) | 2 | 4 |
| CYP2C9 Inhibition (recombinant, MFC substrate) | 0 | 5 |
| CYP2C19 Inhibition (recombinant, CEC substrate) | 1 | -1 |
| CYP2D6 Inhibition (recombinant, MFC substrate) | 3 | 6 |
| CYP2E1 Inhibition (recombinant, EC substrate) | 10 | 7 |
| CYP3A4 Inhibition (recombinant, BFC substrate) | - 7 | -7 |
| CYP3A4 Inhibition (recombinant, BzRes substrate) | 2 | 5 |
| CYP3A5 Inhibition (recombinant, BFC substrate) | - 3 | -10 |

Note: Data were obtained from duplicate determinations
