# Supplementary material for: Effects of a Novel Pharmacologic Inhibitor of Myeloperoxidase in a Mouse Atherosclerosis Model
Source: PLoS One. 2012 Dec 10;7(12):e50767. doi: 10.1371/journal.pone.0050767 (PMC3519467; doi:10.1371/journal.pone.0050767)
Supplement: Table S5 — In vitro Toxicity. (DOC) [file pone.0050767.s006.doc]

Table S5. In vitro Toxicity

| Assay | Test Concentration  (μM) | % Inhibition | IC50 (μM) |
| --- | --- | --- | --- |
| Cell Viability: (HepG2; % inhibition of control values ) | 1  30  100 | 2  6  4 | 4152.5 # |
| Cardiac Toxicity  hERG (automated patch-clamp; % inhibition of tail current) | 0.1  1  10 | 11.1  19.2  25.1 | 32.2 # |

Note: Data were obtained from duplicate determinations.

#: Standard Curve Calculations.
