# Supplementary material for: Effects of a Novel Pharmacologic Inhibitor of Myeloperoxidase in a Mouse Atherosclerosis Model
Source: PLoS One. 2012 Dec 10;7(12):e50767. doi: 10.1371/journal.pone.0050767 (PMC3519467; doi:10.1371/journal.pone.0050767)
Supplement: Table S6 — Pharmacokinetic Parameters in Rats venously or orally administered with INV-315. (DOC) [file pone.0050767.s007.doc]

Table S6. Pharmacokinetic Parameters in Rats venously or orally administered with INV-315

| Dose | PK Parameters (Mean ± SEM) | | | | | | | | |
| --- | --- | --- | --- | --- | --- | --- | --- | --- | --- |
|  | T1/2  (min) | CL  (ml/min/kg) | Vz  (ml/kg) | Vss  (ml/kg) | Bioavail-  ability (%) | Tmax  (min) | Cmax  (ng/ml) | AUClast  (min*ng/ml) | AUCinf  (min*ng/ml) |
|  |  |  |  |  |  |  |  |  |  |
| INV-315  1mg/kg, I.V | 119±84 | 26±2 | 4241±  2958 | 1335±  584 |  |  |  | 39454±2978 | 39660±2996 |
| INV-315  5 mg/kg, PO | 42±4 |  |  |  | 29±1 | 30±0 | 476±4 | 58222±2036 | 58319±2036 |

Note: Pharmacokinetic parameters were derived from non-compartmental model using WinNonlin 5.2.

T1/2 = Elimination half-life, CL = Total body clearance, Vz = Volume of distribution based on the terminal phase, Vss = An estimate of the volume of distribution at steady state, Tmax = Time of maximum observed concentration, Cmax = Concentration corresponding to Tmax, AUClast = Area under the concentration-time curve from the time of dosing to the time of last observation, AUCINF = Area under the concentration-time curve from the time of dosing, extrapolated to infinity*, *Linear/Log trapezoidal method was used for AUC calculation, The bioavailability was calculated based on AUCinf or AUClas
