# Supplementary material for: Effects of a Novel Pharmacologic Inhibitor of Myeloperoxidase in a Mouse Atherosclerosis Model
Source: PLoS One. 2012 Dec 10;7(12):e50767. doi: 10.1371/journal.pone.0050767 (PMC3519467; doi:10.1371/journal.pone.0050767)
Supplement: Table S7 — Metabolic parameters at the end of the treatment period. (DOC) [file pone.0050767.s008.doc]

Table S7. Metabolic parameters at the end of the treatment period

|  | Control | INV-315 low | INV-315 hi |
| --- | --- | --- | --- |
| Body weight (g, before treatment) | 20.8 ± 0.6 | 22.8 ± 0.4 | 21.5 ± 0.2 |
| Body weight (g, after treatment) | 34.4 ± 0.8 | 36.2 ± 1.1 | 34.2 ± 0.4 |
| Mean blood pressure (mmHg) | 88.71 ± 2.4 | 86.42 ± 2.1 | 85.50 ± 2.1 |
| Pulse (beats/min)  Post IPGTT (AUC) (g*min/dL  HDL (mg/dL)  Cholesterol (mg/dL)  Triglyceride (mg/dL) | 580.0 ± 12.3  22.9 ± 1.2  18.7 ± 0.7  1203.7 ± 59.3  117.3 ± 4.1 | 626.8 ± 11.3  22.8 ± 1.7  16.3± 0.6  1391.2 ± 25.4  115.7 ± 1.6 | 621.0 ± 12.4  23.3 ± 1.6  14.9 ± 1.0  1271.2 ± 24.6  109.8 ± 2.3 |

AUC = Area Under Curve; IPGTT = Intra-Peritoneal Glucose Tolerance Test; HDL = High-density Lipoprotein, LDL = Low-density Lipoprotein; Values are expressed as mean ± S.E.M of 9 different mice.
