# Supplementary material for: Effects of a Novel Pharmacologic Inhibitor of Myeloperoxidase in a Mouse Atherosclerosis Model
Source: PLoS One. 2012 Dec 10;7(12):e50767. doi: 10.1371/journal.pone.0050767 (PMC3519467; doi:10.1371/journal.pone.0050767)
Supplement: Table S8 — EC50 and Emax (%) values for vascular response. (DOC) [file pone.0050767.s009.doc]

Table S8. EC_50_ and Emax (%) values for vascular response

| Group | EC_50_(Mol/L) | Emax (%) |
| --- | --- | --- |
| **ACh-induced relaxation** |  |  |
| CONTROL | -7.48 ± 0.14 | 42.3 ± 6.9 |
| INV-315 low | -7.74 ± 0.18 | 48.9 ± 11.0 |
| INV-315 hi | -7.77 ± 0.2 | 60.3 ± 5.8 |
|  |  |  |
| **SNP-induced relaxation** |  |  |
| Control | -7.70 ± 0.05 | 88.2 ± 1.9 |
| INV-315 low | -7.91 ± 0.07 | 91.7 ± 2.0 |
| INV-315 hi | -7.90 ± 0.10 | 94.1 ± 3.0 |
| **Phe-induced contraction** |  |  |
| Control | -5.7± 0.14 | 183.7 ± 25.7 |
| INV-315 low | -5.4 ± 0.07 | 176.2 ± 10.7 |
| INV-315 hi | -5.3 ± 0.11 | 164.2 ± 18.8 |
| L-NAME+Control | -5.7 ± 0.11 | 235.9 ± 20.8 |
| L-NAME+INV 315-low | -5.5 ± 0.18 | 249.1 ± 14.2 |
| L-NAME+INV 315-hi | -5.6 ± 0.06 | 233.7 ± 9.9 |

All values are expressed as mean ± S.E.M, n=5-6.
