# Supplementary figures and images for: Effects of a Novel Pharmacologic Inhibitor of Myeloperoxidase in a Mouse Atherosclerosis Model
Source: PLoS One. 2012 Dec 10;7(12):e50767. doi: 10.1371/journal.pone.0050767 (PMC3519467; doi:10.1371/journal.pone.0050767)

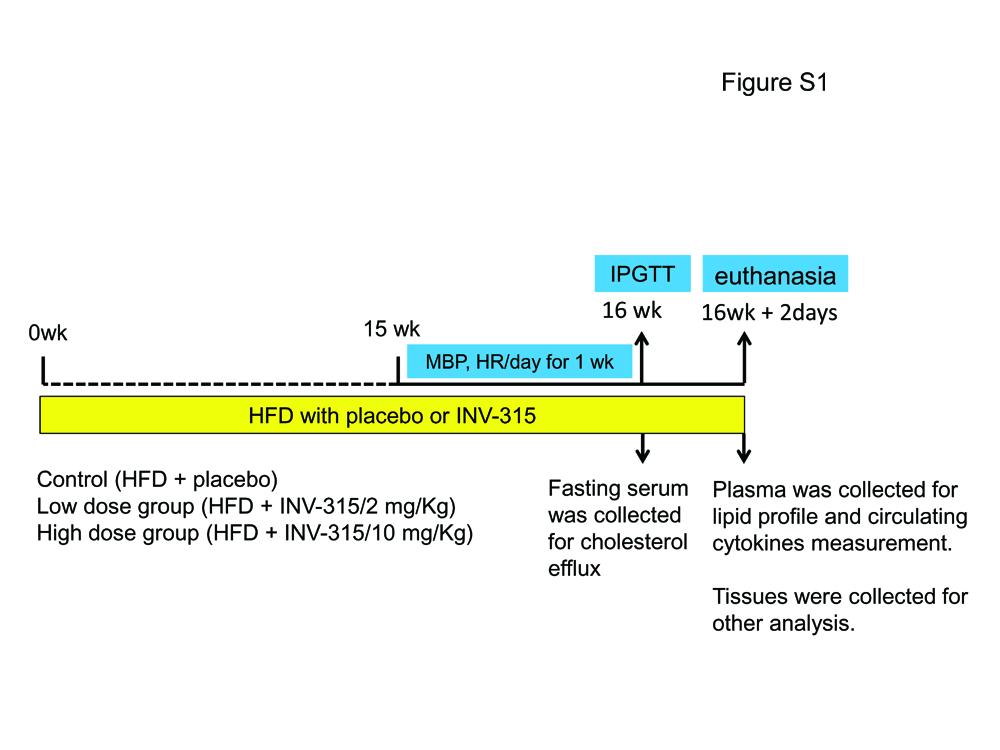

Supplement: Figure S1 — The time line of events of the treatment protocol. (TIF) [file pone.0050767.s010.tif]

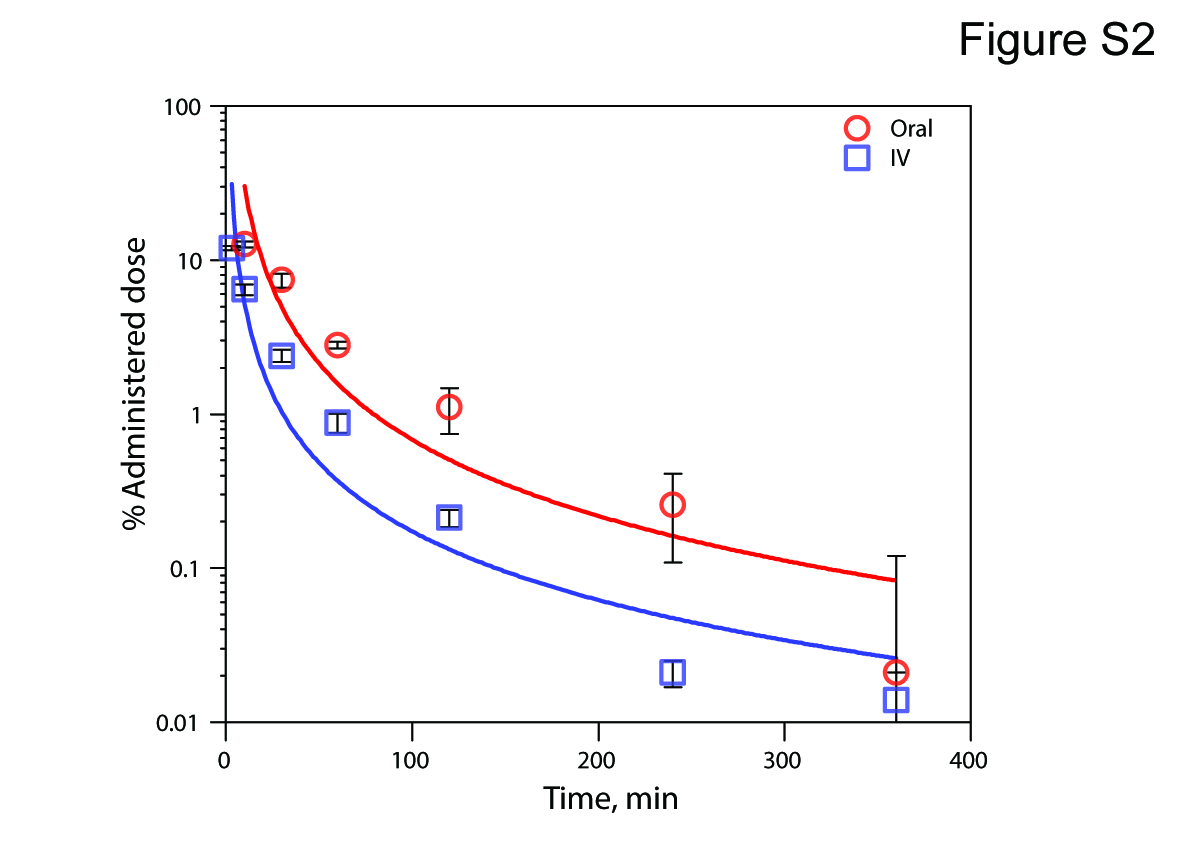

Supplement: Figure S2 — Plasma concentrations of INV-315 after intravenously at 1 mg/kg and orally at 5 mg/kg administration. (TIF) [file pone.0050767.s011.tif]

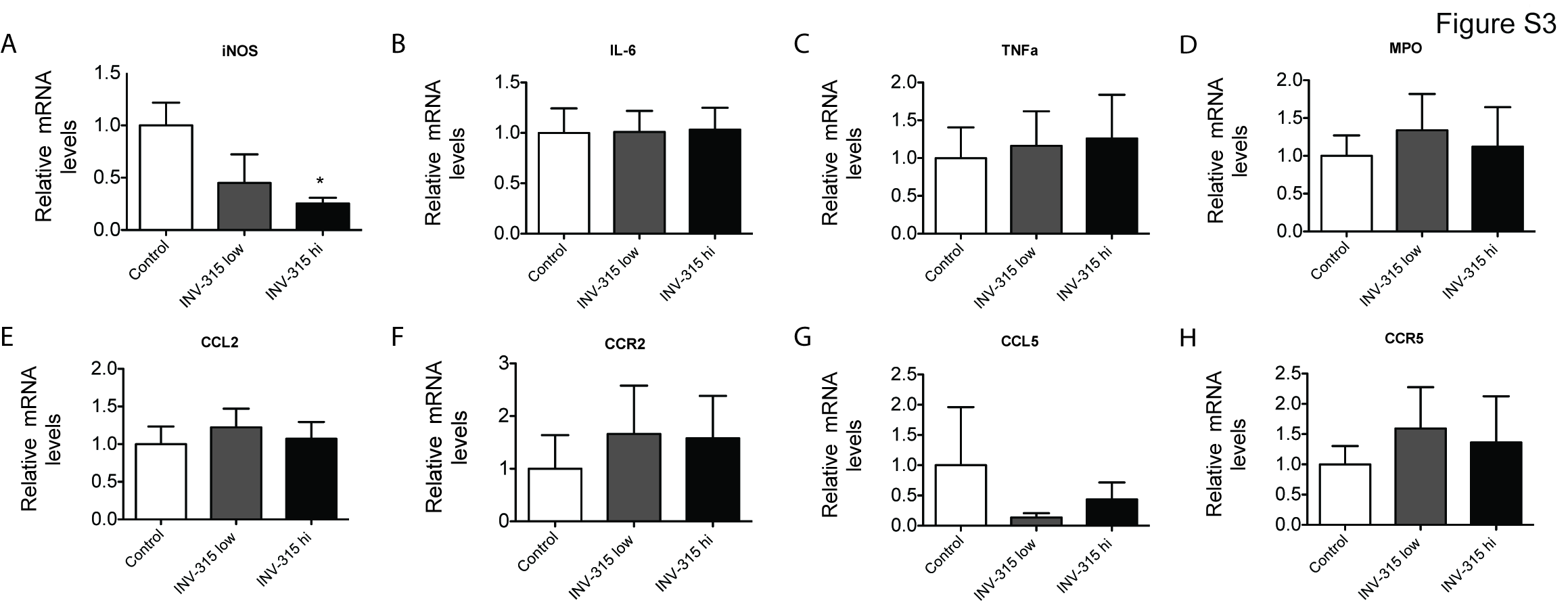

Supplement: Figure S3 — Effects of MPO inhibition on pro-inflammatory gene expression in aorta from ApoE−/− mice fed a HFD. (TIF) [file pone.0050767.s012.tif]

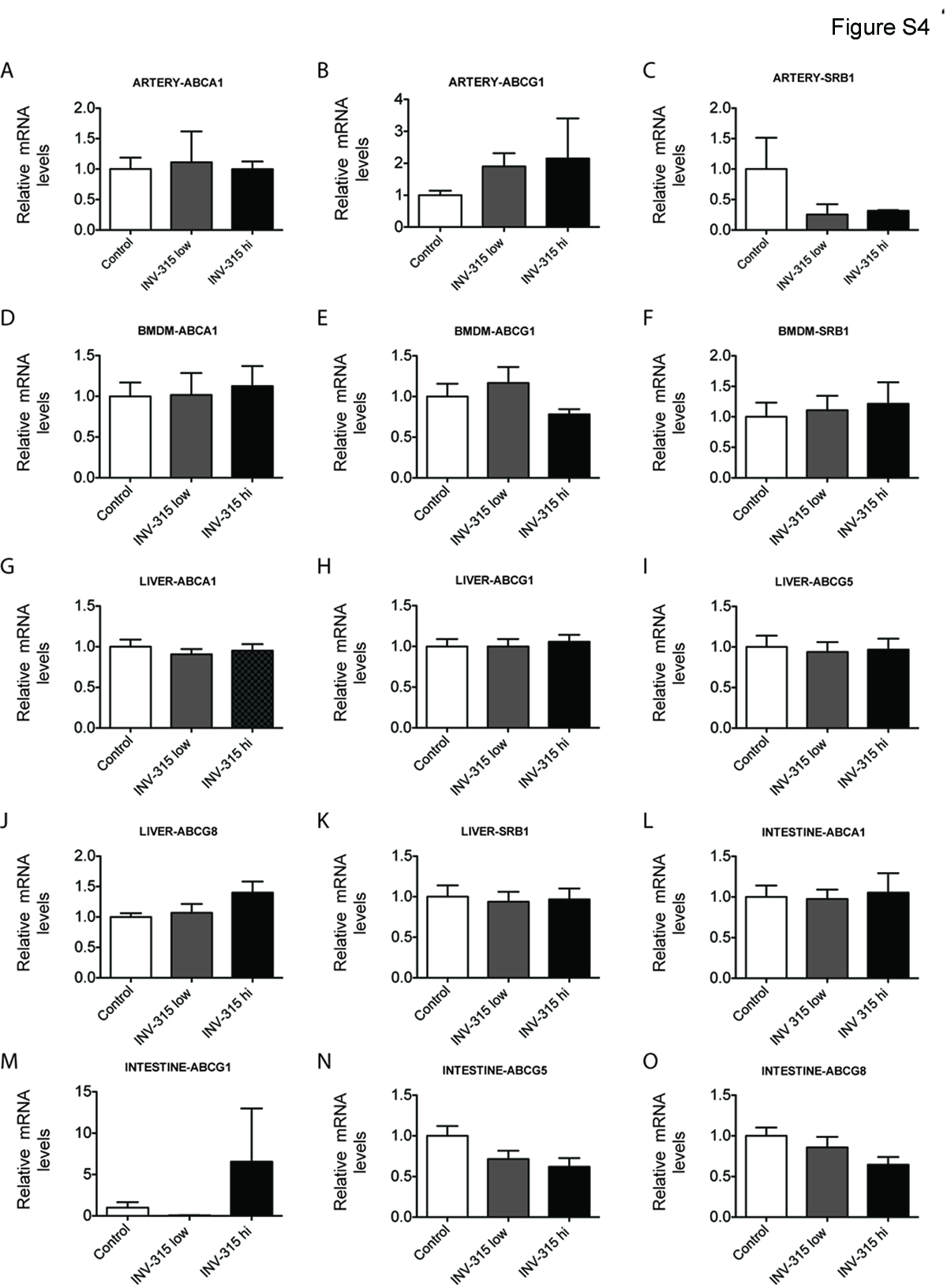

Supplement: Figure S4 — Effects of MPO inhibition on RCT-related gene profiles expression in different tissues from ApoE−/− mice fed a HFD. (TIF) [file pone.0050767.s013.tif]

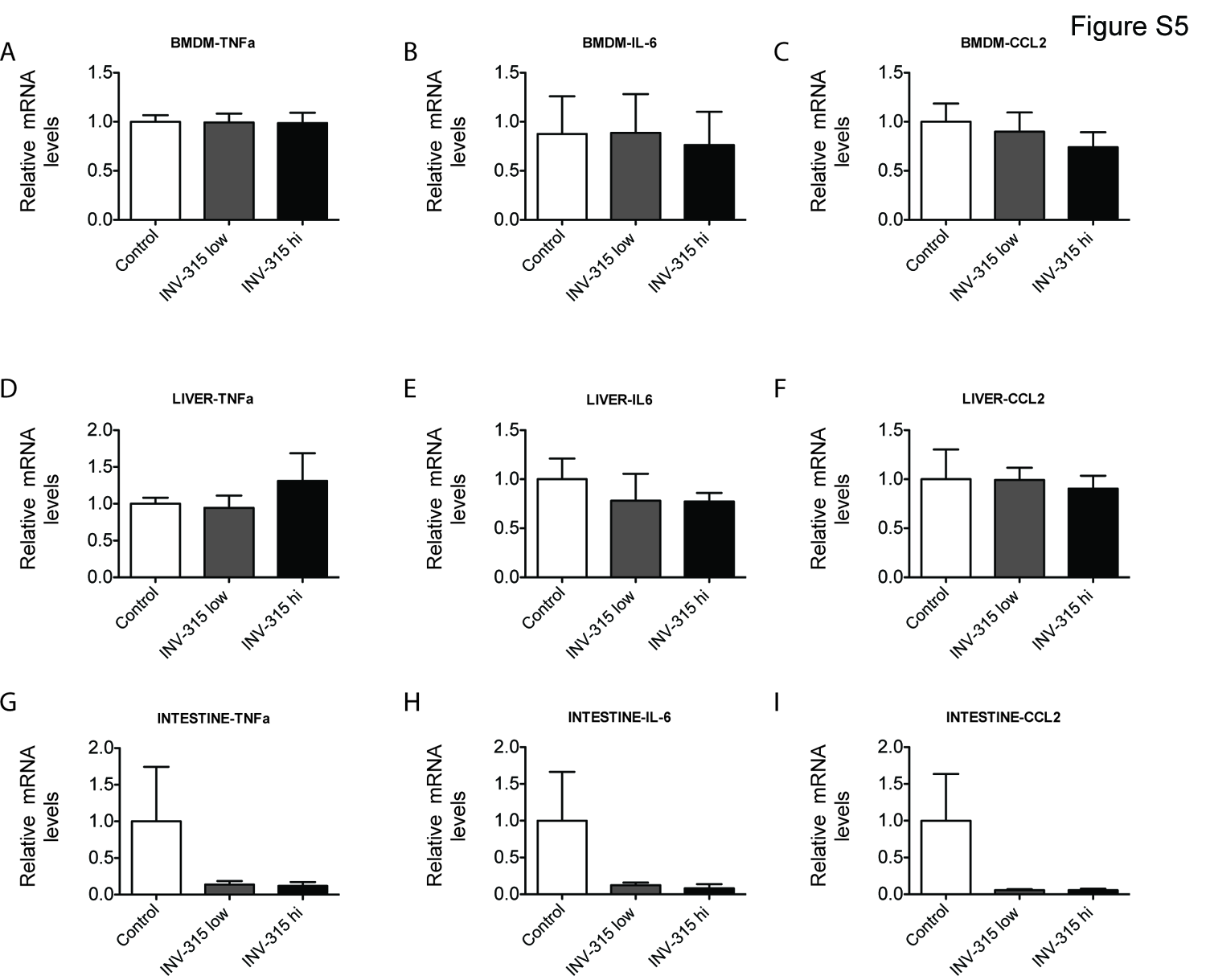

Supplement: Figure S5 — Effects of MPO inhibition on pro-inflammatory gene expression in different tissues from ApoE−/− mice fed a HFD. (TIF) [file pone.0050767.s014.tif]
